# Waterpipe Tobacco Smoking in Turkey: Policy Implications and Trends from the Global Adult Tobacco Survey (GATS)

**DOI:** 10.3390/ijerph121215004

**Published:** 2015-12-08

**Authors:** Cevdet Erdöl, Toker Ergüder, Jeremy Morton, Krishna Palipudi, Prakash Gupta, Samira Asma

**Affiliations:** 1 Rector of Health Science University, Istanbul 34688, Turkey; cevdeterdol@gmail.com; 2 Former Head of the Commission on Health, Family, Labour and Social Affairs of the Grand National Assembly of Turkey, Ankara 06543, Turkey; 3 World Health Organization Country Office, Ankara 06610, Turkey; ergudert@euro.who.int; 4 Centers for Disease Control and Prevention, Atlanta, GA 30341, USA; kpalipudi@cdc.gov (K.P.); sasma@cdc.gov (S.A.); 5 Healis Sekhsaria Institute for Public Health, Navi Mumbai 400701, India; pcgupta@healis.org

**Keywords:** waterpipe, shisha, tobacco smoking, Turkey

## Abstract

Waterpipe tobacco smoking (WTS) is an emerging tobacco product globally, especially among adolescents and young adults who may perceive WTS as a safe alternative to smoking cigarettes. Monitoring the use of WTS in Turkey in relation to the tobacco control policy context is important to ensure that WTS does not become a major public health issue in Turkey. The Global Adult Tobacco Survey (GATS) was conducted in Turkey in 2008 and was repeated in 2012. GATS provided prevalence estimates on current WTS and change over time. Other indicators of WTS were also obtained, such as age of initiation and location of use. Among persons aged 15 and older in Turkey, the current prevalence of WTS decreased from 2.3% in 2008 to 0.8% in 2012, representing a 65% relative decline. Among males, WTS decreased from 4.0% to 1.1% (72% relative decline). While the overall smoking prevalence decreased among females, there was no change in the rate of WTS (0.7% in 2008 *vs.* 0.5% in 2012), though the WTS prevalence rate was already low in 2008. Comprehensive tobacco control efforts have been successful in reducing the overall smoking prevalence in Turkey, which includes the reduction of cigarette smoking and WTS. However, it is important to continue monitoring the use of waterpipes in Turkey and targeting tobacco control efforts to certain groups that may be vulnerable to future WTS marketing (e.g., youth, women).

## 1. Introduction

Tobacco use continues to be the leading preventable cause of morbidity and mortality worldwide because an estimated six million deaths each year are attributed to tobacco use [1,2]. While cigarette smoking remains the main tobacco killer worldwide, for many people, especially youth, tobacco use and addiction is maintained by means other than cigarettes [3]. A way of smoking tobacco that has been highly promoted in recent years is waterpipe tobacco smoking (WTS), also known as narghile, hookah and shisha smoking. The waterpipe in particular is widely used in the Middle East and the Turkish world, and is being marketed as a way of consuming tobacco in Europe [4]. The World Health Organization (WHO) and other authorities have specified that WTS is a public health problem [5].

Many factors may have contributed to the recent WTS spread including socialization, relaxation, pleasure, fashion, curiosity, entertainment, the introduction of sweetened/flavored tobacco, its reduced-harm perception, thriving café culture, mass media, and the internet [3]. Many toxic agents, including carcinogens, heavy metals, other particulate matter, and high levels of nicotine, are efficiently delivered through waterpipe use [6]. A systematic meta-analysis of published studies concluded that WTS is possibly associated with lung cancer, respiratory illness, low birth-weight, and periodontal disease [7].

Many users, however, believe that waterpipe smoke is far less harmful than cigarette smoke because the smoke passes through water, which they presume acts as a filter. Unfortunately, the water only acts as a cooling agent, not as a filter for nicotine, tar, or carcinogens. Furthermore, the cooling process forces the smoker to inhale much deeper, causing the smoke to penetrate deeper into the lungs [6,8,9].

In the Middle East region, WTS is an important public health problem. The region may witness an increase in smoking levels and consequent increase in tobacco-related morbidity and mortality in the future unless intervention measures are initiated now [10]. Thus, for Turkey, it is important to continually monitor the rates of tobacco use and specifically WTS to ensure appropriate measures are taken to stop the tobacco epidemic. The purpose of this paper is to present data on WTS in Turkey from 2008 to 2012, in the context of the tobacco control policies that have been implemented.

## 2. Experimental Section

### 2.1. Tobacco Control Policies

Turkey has made substantial progress in tobacco control in a short time. It ratified the WHO Framework Convention for Tobacco Control (WHO FCTC) in 2004, and substantially amended a 1996 law in 2008 to become one of the most advanced tobacco control laws in the world, with the goal to reduce tobacco consumption and protect citizens from tobacco and waterpipe tobacco use, emphasizing future generations.

Following the ratification of the Convention by Turkey, a National Tobacco Control Programme and Action Plan for 2008–2012 was prepared in line with the WHO FCTC and MPOWER policies and interventions to plan future activities, control tobacco and waterpipe use, and thus protect public health, in particular the health of young people. Table 1 provides the key milestones for tobacco control in Turkey. 

**Table 1:** Key Milestones on Tobacco Control in Turkey.

Date	Milestone
1996	First tobacco control Law 4207 on Prevention of Harms of Tobacco Products
November 2004	Turkey ratified WHO Framework Convention on Tobacco Control (FCTC)
May 2008	Bill amending the law on prevention of hazards of tobacco products of 1996 [11]
July 2009	100% smoke-free law implemented including hospitality sectors.
May 2010	Health warnings on cigarette packages (combined text and graphic warnings)
October 2010	Smoking cessation service launched including a quitline and free distribution of medications.
October 2011	Increase of tobacco excise taxes for tobacco products (80.5% of retail price); 84.2% by January 2014.
July 2012	Total ban on advertisement (including brand sharing and brand stretching) and increase of pictorial health warnings to at least 65% of both sides.

In 2009, the use of all kinds of tobacco products (meaning products that are entirely or partly made of the tobacco leaf as raw material, manufactured to be used for smoking, sucking, chewing, or inhaling through the nose) was banned in all enclosed public places, including water pipe cafes. In order to circumvent the new law, waterpipe cafes started to offer some herbal or aromatic waterpipe products that they claimed did not include tobacco leafs. Law No. 4207, therefore, does not cover waterpipe cafes, and aromatic waterpipes can be smoked in enclosed areas. In addition, it was very difficult to analyze or differentiate whether or not these products contain tobacco leafs during smoke-free inspections. To avoid use of aromatic waterpipe at enclosed public places, the definition of tobacco products has been changed on 24 May 2013 by Law No. 6487/Art. 26 to state that “any waterpipe and cigarettes which do not contain tobacco but used in a way to imitate the tobacco product shall be considered as a tobacco product.” With this change, smoking aromatic waterpipe is banned in waterpipe cafes, as is selling or offering tobacco products, waterpipe, and similar products, whether or not they contain tobacco, for consumption to minors under 18 years old. Law No. 4733 mandates that those who produce, sell, or offer for sale snuff, chewing tobacco, waterpipe tobacco, leaf cigarette paper, or smoking tubes without the certificate of conformity granted by the Authority shall be punished by an administrative fine of 5000 Turkish Lira (about 2500 United States dollars).

**Figure 1:**
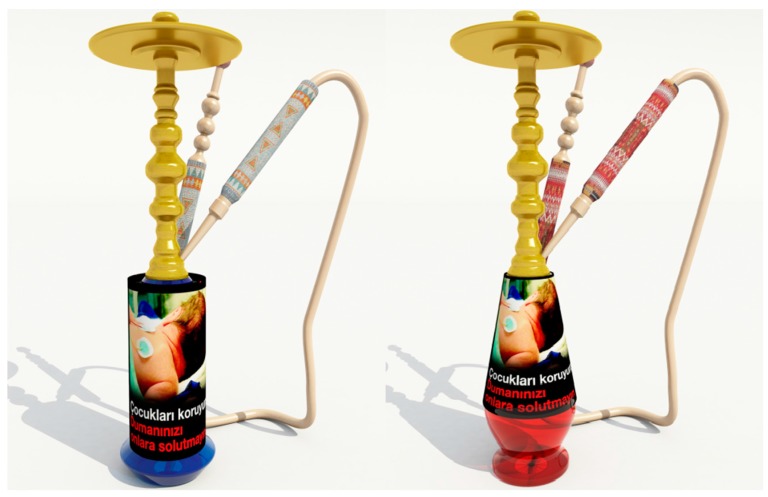
Health warning labels on waterpipes in Turkey.

As with cigarette packages in Turkey, there are two general and one combined health warning labels placed on the waterpipe bowls used for consuming waterpipe tobacco products, covering at least 65% of the principal display area, excluding the bottom (see Figure 1).

Another amendment was introduced in 2013 in order to protect young generations from WTS and to restrict accessibility of waterpipe to minors. Workplace area(s) where waterpipe tobacco products are smoked must have a door-to-door distance of at least 100 m from formal education institutions, tutoring centers, and student dormitories. These areas must not be located within the standalone buildings, premises, and gardens of primary, secondary, and higher education institutions, including preschool education institutions, tutoring centers, private education and training institutions, and cultural and social service buildings.

### 2.2. Methodology and Analysis

In order to measure the prevalence of WTS and the effects of Turkey’s tobacco control policies, data were analyzed from the Global Adult Tobacco Survey (GATS). GATS is a global standard for monitoring tobacco use and key tobacco control indicators. GATS is a nationally representative household survey that uses a standard questionnaire, sample design, and data collection and management procedures [12]. GATS was conducted in Turkey in 2008 (9030 completed interviews; overall response rate 90.9%) and repeated again in 2012 (9851 completed interviews; overall response rate 90.1%). The questionnaire collected data on the current prevalence of WTS (daily or less than daily use), the frequency of WTS (number of waterpipe sessions per day or per week), and the age of initiation and location of use among current waterpipe smokers. Further details about the methodology of GATS Turkey 2008 and 2012 can be obtained elsewhere [13,14].

The relative change of tobacco smoking and specifically waterpipe tobacco smoking from 2008 to 2012 was analyzed. A two-sample t-test was used to indicate the statistical significance of the estimates for comparison between the 2008 and 2012 data.

## 3. Results

Table 2 provides the prevalence rates among persons aged 15 years or older for tobacco smoking, cigarette smoking, and WTS from GATS Turkey 2008 and GATS Turkey 2012.

**Table 2:** Percentage of adults ≥15 years old who are current smokers of cigarettes and waterpipes, by selected demographic characteristics—GATS Turkey, 2008 and 2012.

Demographic Characteristics	2008			2012			Relative Change ^1^		
	Any Smoked Tobacco Product	Any Cigarette ^2^	Waterpipe	Any Smoked Tobacco Product	Any Cigarette ^2^	Waterpipe	Any Smoked Tobacco Product	Any Cigarette ^2^	Waterpipe
	Percentage (95% CI)			Percentage (95% CI)			Percentage		
Overall	31.2 (30.0, 32.6)	31.1 (29.9, 32.5)	2.3 (1.8, 2.9)	27.1 (25.8, 28.3)	26.9 (25.7, 28.2)	0.8 (0.6, 1.1)	−13.4 ^+^	−13.5 ^+^	−64.9 ^+^
Gender									
Male	47.9 (45.9, 50.0)	47.8 (45.7, 49.9)	4.0 (3.0, 5.1)	41.5 (39.4, 43.5)	41.3 (39.3, 43.4)	1.1 (0.7, 1.7)	−13.5 ^+^	−13.5 ^+^	−71.9 ^+^
Female	15.2 (14.0, 16.5)	15.1 (13.9, 16.4)	0.7 (0.4, 1.1)	13.1 (12.0, 14.3)	13.0 (11.9, 14.2)	0.5 (0.3, 0.9)	−13.7 ^+^	−14.0 ^+^	−26.7
Age (years)									
15–24	25.3 (22.2, 28.6)	25.2 (22.2, 28.5)	4.3 (2.9, 6.4)	20.0 (17.4, 22.9)	19.7 (17.1, 22.6)	1.5 (0.9, 2.5)	−20.8 ^+^	−21.8 ^+^	−64.7 ^+^
25–44	39.9 (38.0, 41.9)	39.9 (37.9, 41.8)	2.5 (1.9, 3.3)	35.7 (33.8, 37.5)	35.6 (33.8, 37.5)	1.0 (0.7, 1.6)	−10.7 ^+^	−10.7 ^+^	−59.5 ^+^
45–64	29.5 (27.4, 31.6)	29.4 (27.3, 31.5)	0.9 (0.5, 1.5)	25.9 (23.8, 28.1)	25.8 (23.7, 28.0)	0.1 (0.1, 0.4)	−12.1 ^+^	−12.1 ^+^	−84.7 ^+^
65+	10.3 (8.5, 12.4)	10.0 (8.2, 12.1)	0.2 (0.0, 0.7)	8.8 (7.2, 10.7)	8.8 (7.2, 10.7)	0.0	−14.5	−11.9	
Residence									
Urban	33.0 (31.4, 34.7)	32.9 (31.3, 34.6)	2.9 (2.2, 3.8)	29.0 (27.4, 30.7)	28.9 (27.3, 30.5)	1.0 (0.7, 1.4)	−12.1 ^+^	−12.3 ^+^	−65.8 ^+^
Rural	27.2 (25.3, 29.1)	27.0 (25.2, 29.0)	1.0 (0.6, 1.4)	22.0 (20.4, 23.8)	22.0 (20.3, 23.7)	0.3 (0.2, 0.6)	−18.9 ^+^	−18.7 ^+^	−63.9 ^+^
Education Level									
Not Graduated	15.0 (12.5, 18.0)	14.9 (12.4, 17.9)	0.0 (0.0, 0.2)	11.0 (8.9, 13.4)	11.0 (8.9, 13.4)	0.2 (0.0, 1.6)	−27.0 ^+^	−26.4 ^+^	413.5
Primary	34.0 (32.0, 36.1)	33.9 (31.9, 36.0)	1.4 (0.9, 2.1)	29.7 (27.7, 31.8)	29.7 (27.7, 31.8)	0.3 (0.1, 0.7)	−12.5 ^+^	−12.4 ^+^	−77.7 ^+^
Secondary	31.1 (27.9, 34.4)	31.0 (27.8, 34.3)	2.9 (1.8, 4.5)	27.2 (24.6, 29.9)	27.0 (24.5, 29.7)	0.3 (0.1, 0.9)	−12.5 ^+^	−12.9 ^+^	−88.5 ^+^
High School	40.7 (37.5, 44.1)	40.7 (37.4, 44.0)	5.1 (3.4, 7.5)	33.9 (31.1, 36.8)	33.5 (30.8, 36.4)	2.2 (1.4, 3.4)	−16.8 ^+^	−17.5 ^+^	−56.6 ^+^
University	31.8 (28.1, 35.7)	31.6 (27.9, 35.5)	3.9 (2.4, 6.1)	26.7 (23.5, 30.3)	26.7 (23.4, 30.2)	1.4 (0.8, 2.5)	−15.8 ^+^	−15.5 ^+^	−64.4 ^+^

Current use includes both daily and occasional (less than daily) use; ^1^ Relative change (%) calculated by ((estimate of 2012 − estimate of 2008) / estimate of 2008) **×** 100; ^2^ Includes manufactured cigarettes and hand rolled cigarettes; ^+^ *p* < 0.05.

In 2008, the overall prevalence rate of tobacco smoking was 31.2%. The prevalence of cigarette smoking was 31.1% and WTS was 2.3% (males 4.0%). GATS 2008 results showed a relatively high WTS rate among young adults (4.3% in the group aged 15–24 years) and in urban areas (urban 2.9%; rural 1.0%). It was also relatively high among educated people (high school graduates 5.1%; university graduates 3.9%).

In 2012, the overall prevalence rates of tobacco smoking (27.1%), cigarette smoking (26.9%), and WTS (overall 0.8%; males 1.1%) all declined. The relative reduction of WTS was 64.9%, much higher than the relative reduction of overall tobacco smoking (13.4%) and cigarette smoking (13.5%). This statistically significant decrease in WTS was observed for all demographic subgroups except for women, the elderly (65+), and those with less than primary education; though these groups already had a very low prevalence rate in 2008.

**Figure 2:**
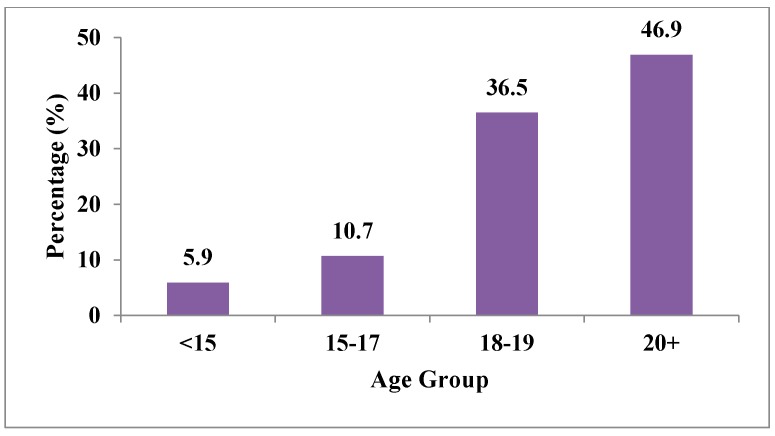
Percentage distribution of age at initiation of waterpipe smoking among current waterpipe users—GATS Turkey, 2012.

**Figure 3:**
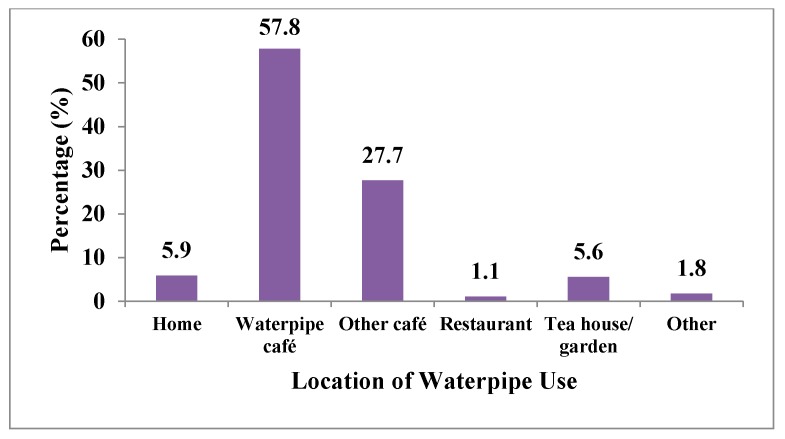
Percentage distribution of location of last waterpipe session among current waterpipe users—GATS Turkey, 2012.

From GATS 2012, the distribution of age (years) of WTS initiation among current waterpipe users is presented in Figure 2. Among this group, 5.9% started smoking waterpipe daily before the age of 15; 10.7% at ages 15–17; 36.5% at ages 18–19; and 46.9% at 20 years or older. This means that more than half of the waterpipe smokers (53.1%) started before the age of 19. Figure 3 presents that more than half of the waterpipe smokers smoked a waterpipe at waterpipe cafes (57.8%), followed by 27.7% at other cafes, 5.9% at home, and 5.6% at tea gardens.

## 4. Discussion

The prevalence rate of WTS declined almost 65% from 2008 to 2012. Prevalence of cigarette smoking, which is the most common form of tobacco use in Turkey, also declined but at a lower rate (13.5% relative reduction). The decline in WTS occurred for most subgroups, which indicates that policies directed at WTS (and smoking in general) are influencing people of various demographic characteristics.

The findings from GATS 2012 suggest that over half of waterpipe smokers started before the age of 19 years. Furthermore, waterpipe users may not be aware of health hazards associated with this behavior. A study carried out in Ankara in 2004 [15] on 273 individuals aged 14–44 years (55% aged 18–24 years) showed that among waterpipe users, 27.1% had no clear idea of the health hazards; 18.3% thought the pipes did no harm to their health; and 27.9% reported that they did not smoke cigarettes, but only a waterpipe.

Global evidence shows a WTS epidemic particularly affecting young people including school and university students [16]. For more than a decade, data from the GYTS have shown that the prevalence of WTS among male and female school students has reached 39.3% and 31.0%, respectively, in some countries in the WHO Eastern Mediterranean Region. A similarly alarming situation is seen among university students. In recent studies, the prevalence among university students was 23%. In the Eastern Mediterranean Region, future projections in some countries suggest that in a few years’ time, cigarette use is likely to decrease while WTS will be on the rise [17]. Furthermore, recent results from the GATS have shown significant use of waterpipe tobacco in European countries such as the Russian Federation (4.4%) and Ukraine (3.2%) [18].

The results of GATS 2008 and 2012 show that the prevalence rate of WTS in Turkey has declined. This suggests that the comprehensive tobacco control policies that have successfully reduced tobacco smoking and particularly cigarette smoking may also have been successful in decreasing smoking of waterpipes. Furthermore, since in 2012 almost 86% of waterpipe smokers smoked waterpipe in cafes, the specific policies that were amended in 2013 aimed at WTS (e.g., restriction of any indoor waterpipe smoking at cafes, regardless of whether tobacco is being used or not; restriction on location of waterpipe cafes near schools) may assist in reducing WTS even further.

However, because there is widespread evidence of waterpipe smoking emerging globally, especially among youth, careful attention should still be paid to ensure that the WTS rate remains or declines from the GATS 2012 levels and that youth are not picking up the behavior. To ensure this, preventive measures encompassing all forms of tobacco smoking should be targeted at adolescents in the school environment and aimed at tobacco use control [19]. People also should be warned about the cancer risk associated with WTS [20]. Education is important to create awareness about the hazards of smoking practices, including waterpipe [21,22]. Because websites may play a role in enhancing or propagating misinformation related to waterpipe smoking, health education and policy measures may be valuable in countering this misinformation [23].

Note that the findings in this paper are subject to a few limitations. All data were self-reported, and social norms (e.g., unacceptability of smoking among women) might have affected responses. Additionally, the data were obtained from household surveys conducted among those 15 years of age and older. Thus, findings among specific vulnerable populations such as younger persons and college students may not be adequately represented.

## 5. Conclusions

Successful implementation of tobacco control policies in Turkey may have reduced WTS as the results in this paper suggest. The decline of both cigarette smoking and WTS provides evidence that comprehensive tobacco control policies work across the spectrum of tobacco products.

However, because of the sufficient scientific evidence showing that WTS is a global public health epidemic and poses serious health threats, it is important to continue focusing on identifying needs and ways in controlling waterpipe tobacco use with a coordinated effort at national, regional, and global levels. Targeted interventions could reduce the prevalence of WTS to even lower levels.

There is a concern about the potential lack of awareness about the harms of waterpipe tobacco use among its users and the public. Furthermore, there is a lack of guidance on how the WHO FCTC should apply to waterpipe tobacco use, such as on the application of pictorial health warnings on waterpipe tobacco packs. Other countries can use the Turkish experience to tackle waterpipe tobacco use in a multisectoral and comprehensive fashion.
